# Short‐ and Long‐Term Growth Response to Multiple Drought Episodes: Evidence of Genetic Adaptation in a Conifer Species

**DOI:** 10.1002/ece3.71398

**Published:** 2025-05-14

**Authors:** Jaime Sebastian‐Azcona, Eduardo P. Cappa, Letitia Da Ros, Blaise Ratcliffe, Charles Chen, Xiaojing Wei, Yang Liu, Shawn D. Mansfield, Andreas Hamann, Yousry A. El‐Kassaby, Barb R. Thomas

**Affiliations:** ^1^ Department of Renewable Resources University of Alberta Edmonton Alberta Canada; ^2^ Irrigation and Crop Ecophysiology Group Instituto de Recursos Naturales y Agrobiología de Sevilla Sevilla Spain; ^3^ Instituto Nacional de Tecnología Agropecuaria (INTA) Instituto de Recursos Biológicos, Centro de Investigación en Recursos Naturales Buenos Aires Argentina; ^4^ Consejo Nacional de Investigaciones Científicas y Técnicas (CONICET) Buenos Aires Argentina; ^5^ Department of Wood Science, Faculty of Forestry University of British Columbia Vancouver British Columbia Canada; ^6^ Department of Forest and Conservation Sciences, Faculty of Forestry University of British Columbia Vancouver British Columbia Canada; ^7^ Department of Biochemistry and Molecular Biology Oklahoma State University Stillwater Oklahoma USA; ^8^ QAAFI & ARC CoE for Plant Success in Nature and Agriculture University of Queensland Brisbane Queensland Australia; ^9^ Department of Botany University of British Columbia Vancouver British Columbia Canada

**Keywords:** dendrochronology, drought, genetics, local adaptation, lodgepole pine, progeny trial

## Abstract

Drought tolerance of tree species is a concern in the context of climate change, and tree ring analyses can be used to assess past growth response(s), to drought events. In the current study, we applied this approach to 1281 individuals with known pedigree in long‐term genetic test plantations of lodgepole pine in western Canada. We assessed resistance, resilience, and recovery metrics, and analyzed their causal relationships with long‐term growth and susceptibility to disease through structural equation modeling. We found that trees with low short‐term resilience to drought events also experienced severe reductions in long‐term growth. Narrow‐sense heritability of drought tolerance metrics was low for short‐term responses at specific sites, while a new long‐term decline index for families showed moderate heritability (${\hat{h}}^{2}$ of 0.15 to 0.30 ± 0.03). We also detected evidence of local adaptation, with trees from lower elevation showing better drought adaptation. We conclude that the selection of genotypes for drought tolerance is possible, and that other species or populations could be screened using this method. We also note that the new long‐term decline index developed in this study shows a higher degree of genetic control than other metrices, and may therefore be of broader interest in dendrochronological research.

## Graphical abstract

Tree ring analysis was applied to 1281 mature lodgepole pine trees growing in four common gardens in central Alberta, Canada. Drought indices were used to assess resistance, resilience, and a structural equation model was used to analyze the relationship between these metrics of drought recovery and susceptibility to disease. Evidence of local adaptation was detected, with trees from lower elevation showing greater drought adaptation. A new long‐term decline index also showed a higher degree of genetic control than other metrics.

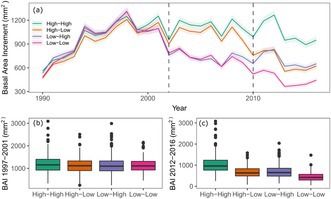

## 1 Introduction

Climate change is expected to cause a steady rise in average temperatures in boreal and temperate regions, combined with increased frequency and severity of extreme drought events (IPCC 2014). This change in climate conditions may outpace the adaptive capacity of tree species, reducing tree vigour, increasing susceptibility to biotic and abiotic stressors, and leading to population decline due to significantly elevated mortality (Martínez‐Vilalta et al. 2012; McDowell et al. 2008). Over the past decades, drought stress has been associated with widespread tree mortality, reduced growth and carbon uptake, in both boreal and temperate forests (Allen et al. 2010; Hogg et al. 2017; Wong and Daniels 2017).

Trees have evolved different drought adaptation mechanisms, often classified as drought tolerance and drought avoidance strategies (Oliveira et al. 2021). Drought tolerance mechanisms involve the development of drought‐resistant xylem tissue, which is able to withstand low water potentials and avoid cavitation (Feng et al. 2021). Anatomically, this is largely due to the production of denser wood, and tracheids with smaller lumen and thicker cell walls, that can reduce the minimum water potential at which the xylem begins to suffer from hydraulic failure (Chave et al. 2009; Hacke et al. 2001). Drought avoidance mechanisms, on the other hand, rely on maintaining higher water potentials in the xylem through early stomatal closure and synthesis of osmoprotective compounds, or investing carbon resources into root production for more efficient water uptake (McDowell et al. 2008). Plastic responses can increase the drought resistance of trees relying on these mechanisms, while other evidence suggests that an individual's phenotypic plasticity is not enough to overcome the increasing drought stress associated with rapidly changing climate in moisture‐limited forest ecosystems (Anderegg et al. 2019). Drought adaptation studies in conifers have shown strong genetic differences between populations, which have been attributed to both local adaptation (George et al. 2017; Housset et al. 2018; Isaac‐Renton et al. 2018) and genetic divergence (George et al. 2017). The genetic control of drought‐adapted traits offers the possibility of selecting specific genotypes that result in more productive and resilient forests.

To select drought‐adapted genotypes, robust metrics of drought adaptation need to be established, and the genetic control of these traits (i.e., their heritability) needs to be determined and verified. Tree rings offer a detailed record of an individual genotype's growth response to drought and other climate and management events (Fritts 1976). The analysis of tree rings, or dendrochronology, has gained prominence in recent years as a tool to study drought response in many tree species (Camarero et al. 2015; Gazol et al. 2018; Serra‐Maluquer et al. 2018; Zang et al. 2014). Two interesting attributes that can be extracted from tree ring analysis are, the ability to maintain growth during a drought event (resistance), and the ability to recover to pre‐drought growth levels after a drought event (resilience, Lloret et al. 2011). These traits have been used to study local adaptation to drought in several conifers (Depardieu et al. 2020; Isaac‐Renton et al. 2018; Montwé et al. 2016), but the question of how both resilience and resistance influence a trees' growth, subsequent to drought events, remains unanswered. While a link between low resilience and increased conifer mortality has been recently observed on a global scale (DeSoto et al. 2020), there is also the hypothesis that a slower recovery from drought stress can indicate the investment of carbon reserves into higher resistant wood, sacrificing more rapid growth (Gessler et al. 2020). Establishing genetic relationships between a short‐term response to drought and long‐term growth can help identify the specific traits that comprise a drought‐adapted tree.

The genetic control of a trees' physiological traits can be studied in common garden experiments, such as progeny test trials. These trials are planted with seedlings originating from seeds collected from different locations and are planted over multiple test sites, where they are all grown under similar environmental conditions. This experimental setting is primarily meant to control environmental effects to reveal genetic differences, quantify phenotypic plasticity, and measure genotype‐by‐environment interactions (de Villemereuil et al. 2016). Most drought adaptation studies, using dendrochronology, focus on a single drought event or a single site (Depardieu et al. 2020; Isaac‐Renton et al. 2018; Montwé et al. 2016). However, studies that include several drought events across multiple test sites have shown significant differences within the same population and between sites in response to drought (Heer et al. 2018; Zas et al. 2020). This variability in drought adaptation traits across different environments emphasizes the importance of analyzing the response to drought across multiple sites to make robust selections of drought‐adapted trees.

Interior lodgepole pine ( *Pinus contorta* Doug. ex Loud. var. *latifolia* Englm.) is a widespread early successional, western North American, conifer species that ranges from latitudes 31 to 64° N and grows under a wide variety of environmental conditions (Burns 1990). It is one of the most important ecological and economic species in western Canada, where multiple genetic test sites were established in the 1970s–1980s. These genetic tests, or common gardens, are now old enough to extract information about growth patterns under different climatic conditions. In the current study, we analyzed both the short‐term responses to drought and long‐term growth of progeny from 40 open‐pollinated families in four 35‐year‐old progeny trials (test sites) in western Canada and explored the effect of environmental conditions and genetic control of drought adaptation.

This study evaluated dendrochronological traits, as drought adaptation proxies, with three specific objectives: (1) develop robust metrics of drought adaptation that describe a genotype's response to drought; (2) investigate causal links among drought responses, disease susceptibility, and long‐term growth potential through structural equation modeling; and (3) determine the degree of genetic control (additive genetic variance) in these drought adaptation traits, and their potential for selection and breeding, across multiple test sites.

## 2 Materials and Methods

### 2.1 Study Site and Plant Material

**FIGURE 1:**
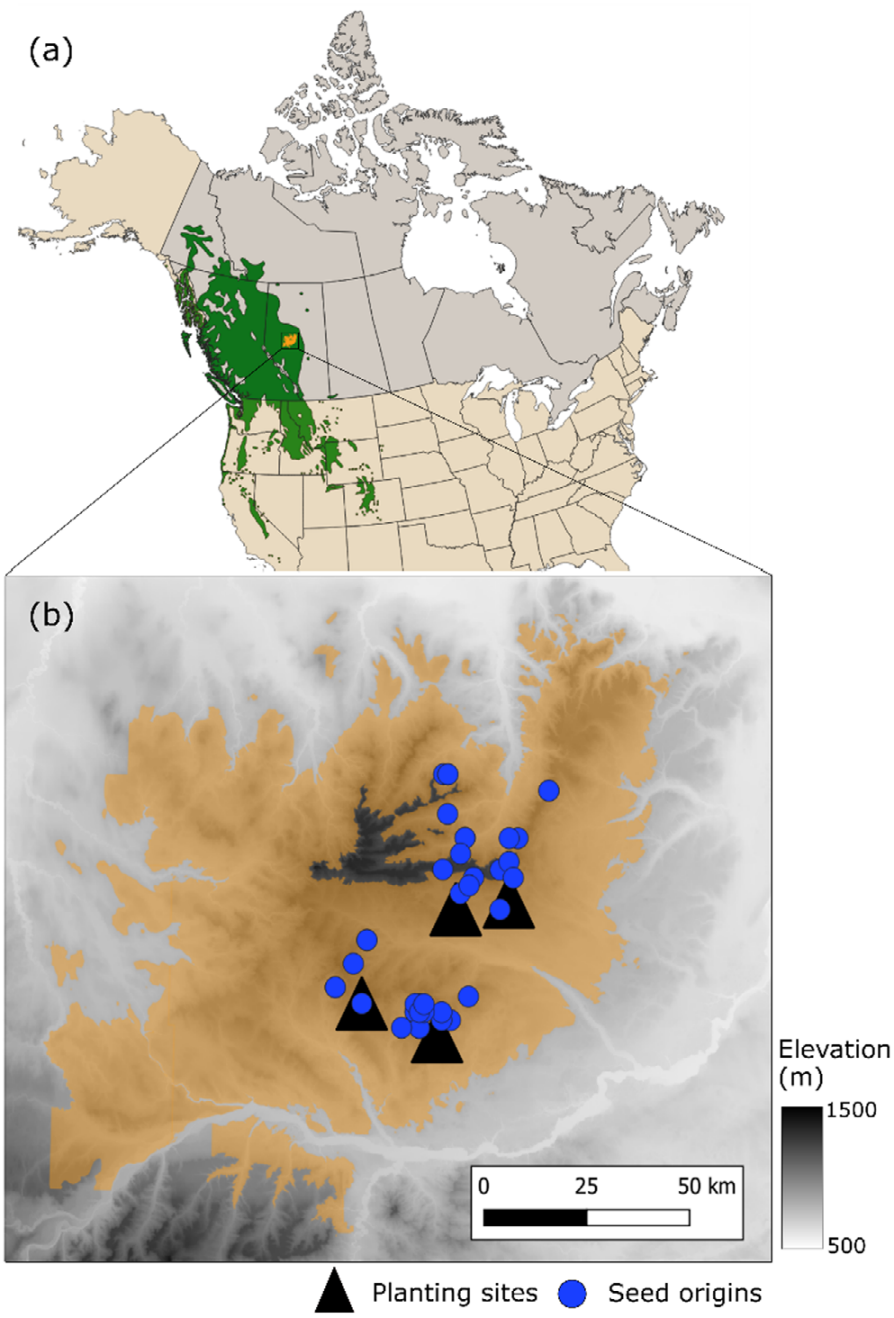
(a) Natural range of lodgepole pine ( *Pinus contorta* ) (light green, interior lodgepole pine, var. *latifolia*, dark green coastal lodgepole pine, var. *contorta*), and Alberta's Region C breeding program area (orange), and (b) parent tree locations (blue dots) and the four progeny test sites (black triangles).

Samples for this study originated from four progeny trials established in 1982 (Alberta, Canada) (Figure 1). These trials were originally planted to test the productivity and adaptability of different open‐pollinated (OP) families within a single breeding program. A subset of families was subsequently selected for a large‐scale genomic selection project focusing on both productivity and resilience to biotic and abiotic stress. At each site, 224 OP families (genetically treated as half‐sib families as only the seed‐donors were known) were divided into 19 sets, and each set was planted randomly in five blocks as four‐tree family row plots. Thus, each OP family was represented by 20 trees per site planted at a 2.5 × 2.5 m spacing.

Since the planting sites and parent tree origins, or provenances, all fall within a single breeding region, there are no major differences in their native climatic conditions, despite some minor elevation differences (Table 1). The study sites range from 1033 to 1127 m in elevation, and averaged from 2.2°C to 2.6°C in mean annual temperature and from 529 to 555 mm in precipitation during the study period (1982–2017). The climatic conditions at the parent tree origins showed greater variability, ranging from 945 to 1280 m in elevation, 1.2°C to 1.9°C in mean annual temperature, and 592 to 718 mm in precipitation, for the climate normal period of 1951–1980. The soil type in three of the sites was luvisolic, with a pH of 5.4 to 5.5, while the fourth site (Judy Creek site; JUDY) had a less evolved and more acidic brunisolic soil with a pH of 3.9. Two of the sites showed unusually high mortality: VIRG (Virginia Hills site) was affected by a wildfire in 1998 killing approximately half of the standing trees, while the higher than normal mortality in JUDY was likely caused by early competition due to insufficient weed control (Table 1). All sites were fenced from the time of establishment to keep out ungulates, and each trial was bordered with one row of trees of the same species to reduce edge effects.

**TABLE 1:** Site, elevation, climate, soil, and mortality information at the four lodgepole pine progeny trial test sites (JUDY = Judy Creek, SWAN = Swan Hills, TIME = Timeau, VIRG = Virginia Hills). The climate variables represent the average over the trial growing period (1982–2016).

Site	Elevation (m)	MAT (°C)	(mm)	CMI (mm)	Soil type	pH	Mortality (%)
JUDY	1097	2.6	529	147	Orthic Dystric Brunisol	3.9	41.8
SWAN	1033	2.3	534	129	Brunisolic Gray Luvisol	5.5	12.6
TIME	1064	2.2	542	143	Brunisolic Gray Luvisol	5.4	17.0
VIRG	1127	2.5	555	180	Brunisolic Gray Luvisol	5.5	57.6

Abbreviations: CMI, climate moisture index; MAP, mean annual precipitation; MAT, mean annual temperature.

From the 224 OP families planted, 40 families, with approximately 10 trees per family per site, were selected for high‐level phenotyping and SNP genotyping (*N* = 1393 trees). In order to sample the extent of the genetic variability within the study, the 40 families were selected based on their 30‐year‐old height breeding values representing high, average, and low productivity families. Trees within families were selected across the progeny trials, that is, not from a single‐row plot of the family (Cappa, Chen, et al. 2022; Cappa, Ratcliffe, et al. 2022). For the selected families and trees within families, total height and diameter at breast height (DBH; 1.3 m) were remeasured at age 35 (in 2017), and western gall rust (*Endocronartium harknessii* Moore) infections present in the trial were recorded at age 36 (in 2018), and scored on a scale from 0 to 6 depending on the severity and location (branches or stem) of the galls. The presence of galls indicates a previous infection, which may have occurred years before the scoring. A competition index was calculated using DBH measurements at age 30 (only the 1393 selected trees were measured at age 35, while all 224 families and their progeny (10,694) were measured at age 30), using the following equation (Contreras et al. 2011; Rouvinen and Kuuluvainen 1997):

(1) $$\sum_{i=1}^{n}\left(\frac{d_{i}}{d}\right)\times \arctan\left(\frac{d_{i}}{{\text{dist}}_{i}}\right)$$

where n = number of neighbour trees within the closest two rows and columns (7.1 m radius) to the cored tree (see dendrochronology analysis below), $d_{i}$ = DBH of the *i*th neighbour tree (cm); d = DBH of the cored tree (cm); and ${\text{dist}}_{i}$ horizontal distance from the *i*th neighbour tree to the cored tree (m). This competition index was used for spatial analysis and to remove the confounding effect of large blocks due to multi‐tree row plots of each family (Cappa, Ratcliffe, et al. 2022).

### 2.2 Dendrochronology Analysis

In June 2017, one north–south (bark‐to‐bark), 5 mm increment core was taken at approximately breast height (1.3 m) from each selected tree (cored tree). Wood density profiles from the north‐half of the tree cores were determined at a resolution of 0.0254 mm, as previously described (Da Ros et al. 2021). We performed a careful quality check on the density profiles, discarding density fluctuations due to cracks, wounds, or other wood imperfections. The average wood density of the whole core was calculated from these density profiles.

Wood density profiles from the north‐half of the core were also used to calculate tree ring width, using the sharp contrast between earlywood and latewood densities to identify annual growth rings. Core sections in which this contrast was not sufficiently clear to determine the width were discarded (mostly in the inner rings that are not perpendicular to the core so that the exact point of transition from one year to the next is not obvious) and these discarded cores represented 8% (112 trees) of the sampled trees (1393). To validate the ring width measurements from the wood density profiles, we used the south‐half of 400 cores (10 families) to directly measure ring width. These cores were sanded using progressively finer grains, scanned at a resolution of 3200 dpi (Epson Perfection V800 Photo), and the tree ring width was measured using WinDENDRO (version 2017a, Regent Instruments Canada Inc.). All ring width measurements were first visually cross‐dated and then checked for errors using COFECHA (Holmes 1983). Ring width values were then used to calculate annual basal area increment (BAI) using the bai.out function in the dplR package for R (Bunn 2008). The results of measurements coming from one and two cores per tree were compared and these comparisons yielded no significant differences, so we assumed that one core accurately represented tree growth.

**FIGURE 2:**
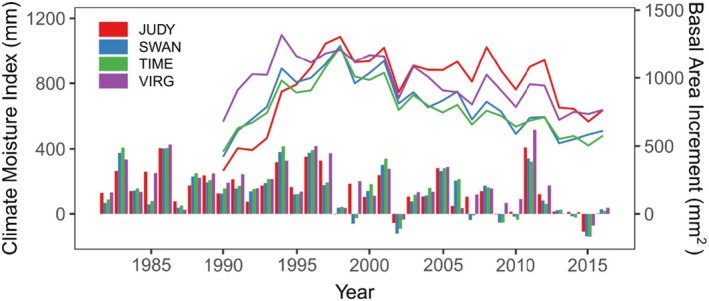
Average yearly basal area increment (lines) based on increment cores taken at 1.3 m height and climate moisture index (bars) at each of the four lodgepole pine progeny trial test sites (JUDY = Judy Creek, SWAN = Swan Hills, TIME = Timeau, VIRG = Virginia Hills) during the trial growing period (1982–2016). Climate moisture index values below 0 are considered drought events, which occurred frequently in the second half of the growing period.

Based on both growth and climate data, we selected two different drought events, where trees showed a clear decrease in growth, to calculate several drought‐response indices (Figure 2). These drought events were characterized by a particularly dry end of the growing season in the previous year (August/September of 2001 and 2009), followed by a very dry summer (2002) and a dry early growing season (April–June, 2010; Figure S1), respectively. Although another drought period occurred in 2014/2015, it was not included in this analysis because, at the time of sample collection in 2017, there were insufficient post‐drought years to quantify growth recovery. We calculated the resistance and resilience indices for each drought event as follows (Lloret et al. 2011):

(2) $$\text{Resistance}=\frac{{\mathrm{BAI}}_{\text{event}}}{{\mathrm{BAI}}_{\mathrm{pre}}}$$

(3) $$\text{Resilience}=\frac{{\mathrm{BAI}}_{\text{post}}}{{\mathrm{BAI}}_{\mathrm{pre}}}$$

where, BAI_event_ = the BAI of the drought event year, BAI_pre_ = average BAI of the four years prior to the drought event, and BAI_post_ = average BAI of the four years following the drought event. We chose four‐year averages as a compromise between having as many years as possible in order to have a good representation of the average growth while avoiding other minor drought events.

Since resilience and resistance represent the short‐term response of a tree to a drought event, we used a similar approach to develop a new simple index that would represent the long‐term response of a tree to repeated drought events. Since the studied trees within these trials are evenly spaced and even aged (i.e., following a common growth pattern), we compared the growth during the five‐year period of maximum productivity, just prior to the first severe drought event (1997–2001), and the growth during the last five years recorded (2012–2016), as follows:

(4) $$\text{Decline}=\frac{{\mathrm{BAI}}_{\max}}{{\mathrm{BAI}}_{\text{last}}}$$

where, BAI_max_ = average BAI during 1997–2001, and BAI_last_ = average BAI between 2012–2016. Trees displaying decline values close to 1 represent non‐declining individuals that were able to maintain a constant growth pattern throughout their lifetime, while trees with larger decline values (> 1) represent individuals that experienced a severe growth reduction compared to their early growth. We chose five‐year averages for this index following the same reasoning explained above, although 2015, a drought year, was included in BAI_last_.

### 2.3 δ^13^C Analysis

We used the two small residual slabs, taken from the full length of the north‐half of each 5 mm core, to perform δ^13^C analysis. Core samples were dried at 65°C for 3 days and ground using a Qiagen Tissuelyser II (Qiagen Inc.‐Canada, Ontario, Canada). The ground wood samples were subsequently sent to the Stable Isotope Lab of InnoTech Alberta (Victoria, BC, Canada) for δ^13^C analysis, without cellulose extraction (Isaac‐Renton et al. 2018; Hu et al. 2021). Samples were analyzed using an established method on a MAT253 mass spectrometer with Conflo IV interface (Thermo Fisher Scientific, Waltham, MA, USA) and a Fisons NA1500 EA (Fisons Instruments, Milano, Italy). The results were normalized and reported against Vienna Pee Dee Belemnite (the established standard for carbon‐13 [δ^13^C] estimates [Craig 1957]).

### 2.4 Climate

We used monthly and annual variables from 1982 to 2016 to represent the climatic conditions that the trees experienced since establishment. To characterize the climate of origin of the parent trees, we extracted the average for the normal period of 1950–1980 using the BioSIM software v.11.3 (Régnière et al. 2017), which is the most recent normal period before the trials were established. Data were then interpolated from the four closest weather stations available and corrected for geographical location and elevation. The variables used in this study were mean annual temperature (MAT), mean annual precipitation (MAP), and climate moisture index (CMI = precipitation – Hargraves evapotranspiration).

### 2.5 Pedigree Reconstruction and Correction

Due to the nature of open‐pollinated seed collections, pedigree verification was necessary using available SNP markers (Cappa, Chen, et al. 2022). Pedigree errors were identified and corrected using a custom R script to compare observed pairwise additive relationship estimates to their expected values (e.g., 0.25 for half‐siblings). Observed pairwise additive relationship estimates were calculated according to VanRaden (2008). The decision to assign a sample to a genetic group (pedigreed family) was based on the samples' maximum average observed relationship to each genetic group (see Figure S2 in Cappa, Ratcliffe, et al. 2022).

### 2.6 Statistical Analyses

Average values of the dendrochronology and growth traits for each site were calculated using mixed models in which the site was considered a fixed effect and the block and set nested within the block were considered random effects. Where possible, only a single tree was sampled from each four‐tree‐row plot; therefore, the plot effects were not fitted. These models were used to calculate the estimated marginal means for each trait, and significant differences between sites were analyzed using pairwise comparisons with a Tukey correction. Linear phenotypic relationships between traits were analyzed with linear models and Pearson's product–moment correlations, and correlation *p*‐values were corrected for multiple comparisons using a Bonferroni adjustment. In all cases, the normality of the data was checked, *a priori*, and either squared root or logarithmic transformations were applied when necessary.

The complex relationships between traits were further analyzed using a structural equation model (SEM). These SEMs are confirmatory analyses that test whether a theoretical model defined through direct and indirect causal relationships between traits can properly explain the variance in the data, while identifying if there are important relationships missing (Lefcheck 2016). In this case, we used a directed acyclic (or piecewise) SEM, drawing on applications from graph theory. This approach enabled us to work with non‐normal and non‐independent data and calculate the bivariate relationships within the model using generalized linear mixed models. Therefore, the relationships in the model were corrected for the random effects of the experimental design (site, block, and set) and genetics (family). The goodness‐of‐fit of the SEM was calculated using Fisher's C statistic that follows a chi‐squared distribution, and a *p*‐value above 0.05 indicated that the model did not miss any significant relationships. Another advantage of the SEM method is that it tests for the effect of all links that are not defined in the original model, thereby identifying significant relationships that were not initially expected. The strength of the individual linear relationships in the model is represented by standardized coefficients (SC) that range between −1 and +1. The SEMs were calculated using the *piecewiseSEM* package for R (Lefcheck 2016).

Narrow‐sense heritability estimates and genetic correlations between traits and sites were calculated from multi‐trait individual‐tree mixed models with the following equation using the Expectation–Maximization algorithm, followed by one round with the Average‐Information algorithm, to compute its standard errors in the *breedR* package for R (Muñoz and Sanchez 2019):

(5) Traits=μ+Provenance+Block+Block:Set+Pedigree+ε

where Traits is a vector containing all the individual tree observations on traits or sites, μ is the fixed effect for the overall mean, Provenance is the fixed effect genetic group to account for the means of the different origins of parents, Block and Block:Set are the random effects of the experimental design, Pedigree is the random additive genetic effects (breeding values, BVs) of parents (mother and father when known by pedigree reconstruction) and offspring, and *ε* is the random residual term. For heritability estimation and trait genetic correlations, a separate multi‐trait model was used for each site, and one model for all the combined data where the Traits vector included the seven traits analyzed (decline, resilience 2002 and 2010, resistance 2002 and 2010, height, and DBH (age 35)). For additive genetic correlations between sites, we defined one multi‐trait model for each trait where the same trait in different sites was considered a different variable, so the Traits vector had four variables, one for each site. Narrow‐sense heritability estimates (${\hat{h}}^{2}$) were calculated as:

(6) $${\hat{h}}^{2}=\frac{{\hat{\sigma }}_{a}^{2}}{{\hat{\sigma }}_{a}^{2}+{\hat{\sigma }}_{\text{Block}}^{2}+{\hat{\sigma }}_{\text{Block}:\mathrm{Set}}^{2}+{\hat{\sigma }}_{\varepsilon }^{2}}$$

where ${\hat{\sigma }}_{a}^{2}$is the estimated additive variance, ${\hat{\sigma }}_{\text{Block}}^{2}$ and ${\hat{\sigma }}_{\text{Block}:\mathrm{Set}}^{2}$ are the estimated variances explained by the experimental design of blocks and sets nested within blocks, and ${\hat{\sigma }}_{\varepsilon }^{2}$ is the estimated residual variance. Additive genetic correlations between traits or sites ($\hat{r}$) were calculated as:

(7) $$\hat{r}=\frac{{\hat{\sigma }}_{a_{\mathrm{ij}}}}{\sqrt{{{\hat{\sigma }}_{a}^{2}}_{\mathrm{ii}}+{{\hat{\sigma }}_{a}^{2}}_{\mathrm{jj}}}}$$

where ${\hat{\sigma }}_{a_{\mathrm{ij}}}$ is the estimated additive covariance between traits or sites *i* and *j*, and ${{\hat{\sigma }}_{a}^{2}}_{\mathrm{ii}}$ and ${{\hat{\sigma }}_{a}^{2}}_{\mathrm{jj}}$ are the estimated additive variances of traits or sites *i* and *j*. The 95% confidence interval for the additive genetic correlations was calculated as two times their standard errors. When this confidence interval did not include zero we considered the additive genetic correlation was statistically different from zero.

We used the same multi‐trait models to calculate family breeding values (BVs), which were then used to explore local adaptation by comparing parent BVs to the parent tree (seed donor) elevation of origin and other climate variables. We observed a stronger relationship between parent BVs and elevation, than with any other climate variables, which may be due to the small overall range of the study area and the low variation in climate variables. Therefore, only linear relationships with elevation are reported in the results.

## 3 Results

### 3.1 Drought Response at Different Test Sites

The drought events in 2001–2002 and 2009–2010 showed a negative impact on tree growth at all four progeny trial sites (Figure 2). Overall, the 2002 drought event appeared to be more significant than the 2010 drought event as the resilience and resistance values were consistently lower in 2002, except for resilience at the JUDY site (Table 2). In addition, there were significant differences between sites in their response to drought. The Swan Hills (SWAN) and Timeau (TIME) sites showed a stronger reduction in growth due to drought, exhibiting generally higher decline and lower resistance and resilience values, compared to the JUDY and VIRG sites (Table 2). These differences were particularly strong in resilience to the 2002 drought event and in the long‐term decline. The SWAN and TIME sites showed the lowest resilience in 2002 (0.78 and 0.75 average at each site, respectively) and the highest decline (1.83 average for both SWAN and TIME), followed by VIRG (1.52 average decline and 0.82 average resilience in 2002), and then JUDY, which was the least affected (1.38 decline and 0.91 resilience 2002). We observed a similar pattern in growth at JUDY, which showed the highest height and the second‐highest DBH (1371 cm and 19.4 cm, respectively). VIRG showed the second highest height and larger DBH (1332 cm and 20.1 cm) and SWAN and TIME showed the lowest values in both traits (1249 cm and 18.3 cm in SWAN, and 1268 and 17.6 in TIME, respectively) (Table 2).

**TABLE 2:** Mean values (±SE) by trait at each planting site (JUDY = Judy Creek, SWAN = Swan Hills, TIME = Timeau, VIRG = Virginia Hills) for the five drought response indices, height and diameter at breast height (DBH, 1.3 m) measured at age 35 years of the tested lodgepole pine trees. Means with the same letter indicate no significant difference between sites at *p* < 0.05, using a Tukey adjustment for multiple comparisons.

Trait	JUDY	SWAN	TIME	VIRG
Decline	1.38 (0.03) a	1.83 (0.04) c	1.83 (0.04) c	1.52 (0.04) b
Resilience 2002	0.91 (0.01) a	0.78 (0.01) bc	0.75 (0.01) c	0.82 (0.01) b
Resilience 2010	0.87 (0.01) b	0.78 (0.01) c	0.86 (0.01) b	0.92 (0.02) a
Resistance 2002	0.75 (0.01) a	0.74 (0.01) ab	0.71 (0.01) c	0.72 (0.01) bc
Resistance 2010	0.83 (0.01) a	0.74 (0.01) b	0.87 (0.01) a	0.87 (0.01) a
Height 35 (cm)	1371 (8.5) a	1249 (7.8) c	1268 (7.9) c	1332 (9.2) b
DBH 35 (cm)	19.4 (0.16) b	18.3 (1.15) c	17.6 (0.15) d	20.1 (0.18) a

**FIGURE 3:**
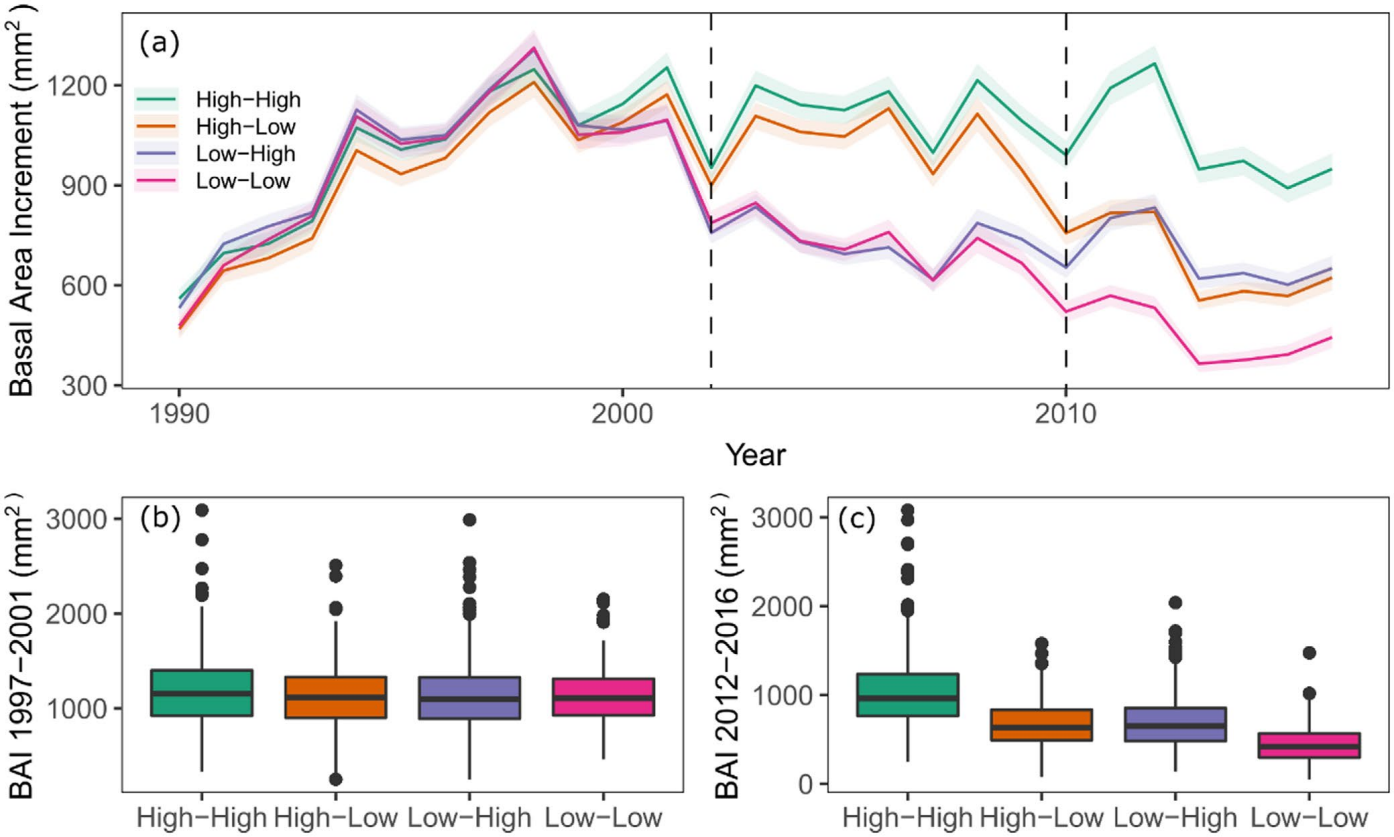
Average yearly basal area increment (BAI) of the lodgepole pine trees at the four progeny trial test sites divided by drought resilience groups (a). The high‐high (*n* = 388) group represents trees that showed good resilience (> 0.8) in both the 2002 and 2010 drought events (dashed vertical lines), high‐low (*n* = 304) and low‐high (*n* = 356) groups showed good resilience in only one drought event, and the low‐low (*n* = 233) group showed poor resilience after both drought events. The boxplots represent the average BAI during the five‐year period of maximum growth before the first severe drought event (1997–2001, b) and the average BAI of the last 5 years of the study period (2012–2016, c).

We tested whether the growth preceding the drought event had an effect on the tree response to drought and found a minor positive relationship between resistance to the 2002 drought event and previous growth (*p* = 0.002), which was stronger at the SWAN (*p* = 0.008) and TIME (*p* = 0.015) sites (Figure S2). These results also showed a significant negative effect of previous growth in resilience to the 2010 drought event (*p* = 0.044), which in this case was stronger in JUDY (*p* = 0.059) and TIME (*p* = 0.080). The other three drought response indices only showed a significant relationship with previous growth at one of the sites, but in all cases, trees with higher growth prior to the drought event were more severely affected by drought stress. At JUDY, trees with higher growth prior to 2002 had lower resilience to the 2002 drought event and higher long‐term decline. At TIME, trees with greater growth prior to the 2010 drought event also showed lower resistance to drought. Growth prior to 2002 also showed no effect on the subsequent resilience to multiple drought events (Figure 3). However, the ability to recover, or not, after one or two drought stress events had a strong effect on the subsequent growth of the studied trees at all four sites (Figure 3, Figure S3). Trees with a high resilience to both drought events were able to maintain their growth rates before and after each drought event (Figure 3b,c).

### 3.2 Drought Response and Long‐Term Growth

**FIGURE 4:**
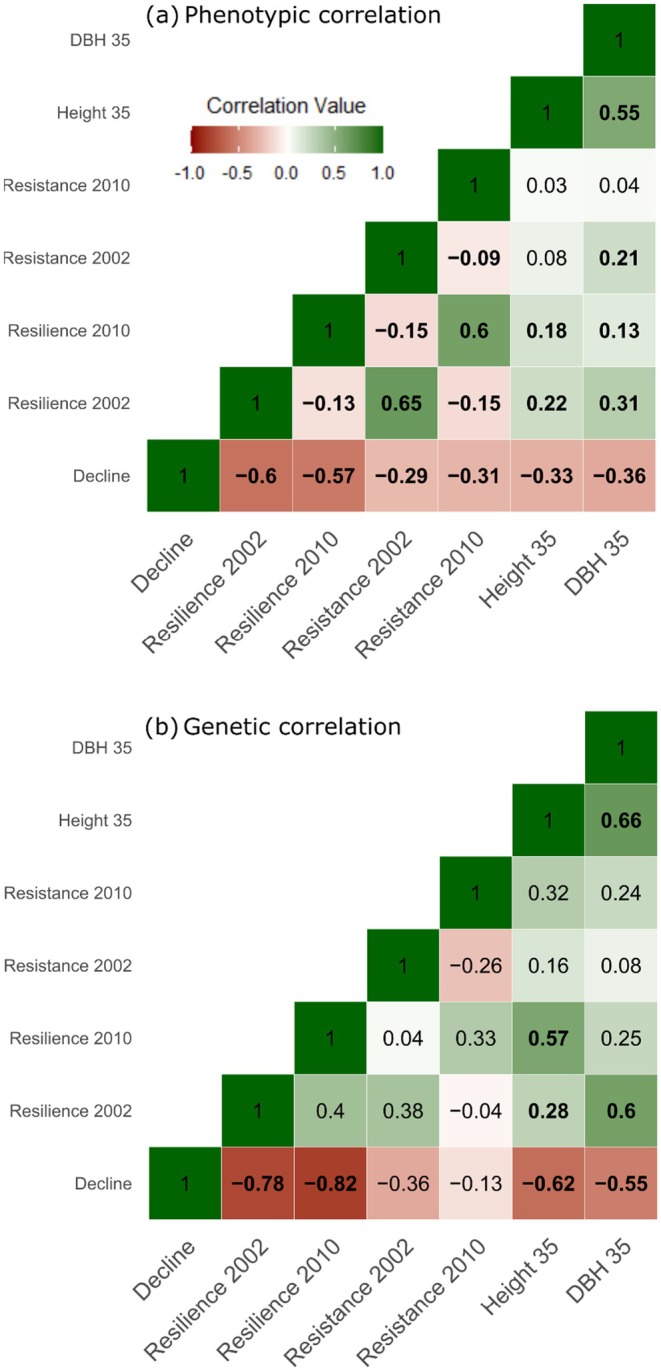
Phenotypic (a) and additive genetic (b) correlations of 40 lodgepole pine families across four progeny trial test sites, between drought response indices, height, and diameter at breast height (DBH) at age 35 years (*N* = 1393). Bold numbers represent significant correlations with Bonferroni adjusted *p*‐values for phenotypic correlations and when the correlation values were higher than two times the standard error for additive genetic correlations.

We analyzed both the phenotypic (Figure 4a) and genetic (Figure 4b) correlations between drought response indices and long‐term performance. The ability of a tree to recover from a drought event, maintaining a growth rate similar to that recorded before the event, had a strong effect on the trees' long‐term growth (Figure 4a). While trees with higher resilience to both the 2002 and 2010 drought events had a significantly lower long‐term decline (*R* = −0.60, *p* < 0.001 and *R* = −0.57, *p* < 0.001, respectively). The ability to maintain growth during a drought event had a lower, but significant, impact on the long‐term success of the tree with correlation values of −0.29 (*p* < 0.001) and −0.31 (*p* < 0.001) between decline and resistance to the 2002 and 2010 drought events, respectively. The two short‐term response indices, resistance and resilience, were highly correlated for the same drought event (*R* = 0.65, *p* < 0.001 in 2002 and *R* = 0.60, *p* < 0.001 in 2010). In contrast, resistance (*R* = −0.09, *p* = 0.003) and resilience (*R* = −0.13, *p* < 0.001), corresponding to different drought events, were negatively correlated (Figure 4a).

Trees that showed a better drought response exhibited higher final growth (Figure 4a). Long‐term decline showed the highest correlations with both height and DBH at age 35 (*R* = −0.33, *p* < 0.001, and *R* = −0.36, *p* < 0.001, respectively). Among the two short‐term indices, resilience, with correlation values from 0.13 to 0.31, had a stronger effect than resistance (0.03–0.21) on the final growth (height and DBH) of the trees after both drought events (Figure 4a). For the two drought events, the 2002 drought showed a stronger effect on the final growth (0.08–0.31) than the 2010 drought event (0.03–0.18, Figure 4) across both resilience and resistance. Furthermore, all phenotypic correlations followed very similar trends across all four sites (Figure S4).

**FIGURE 5:**
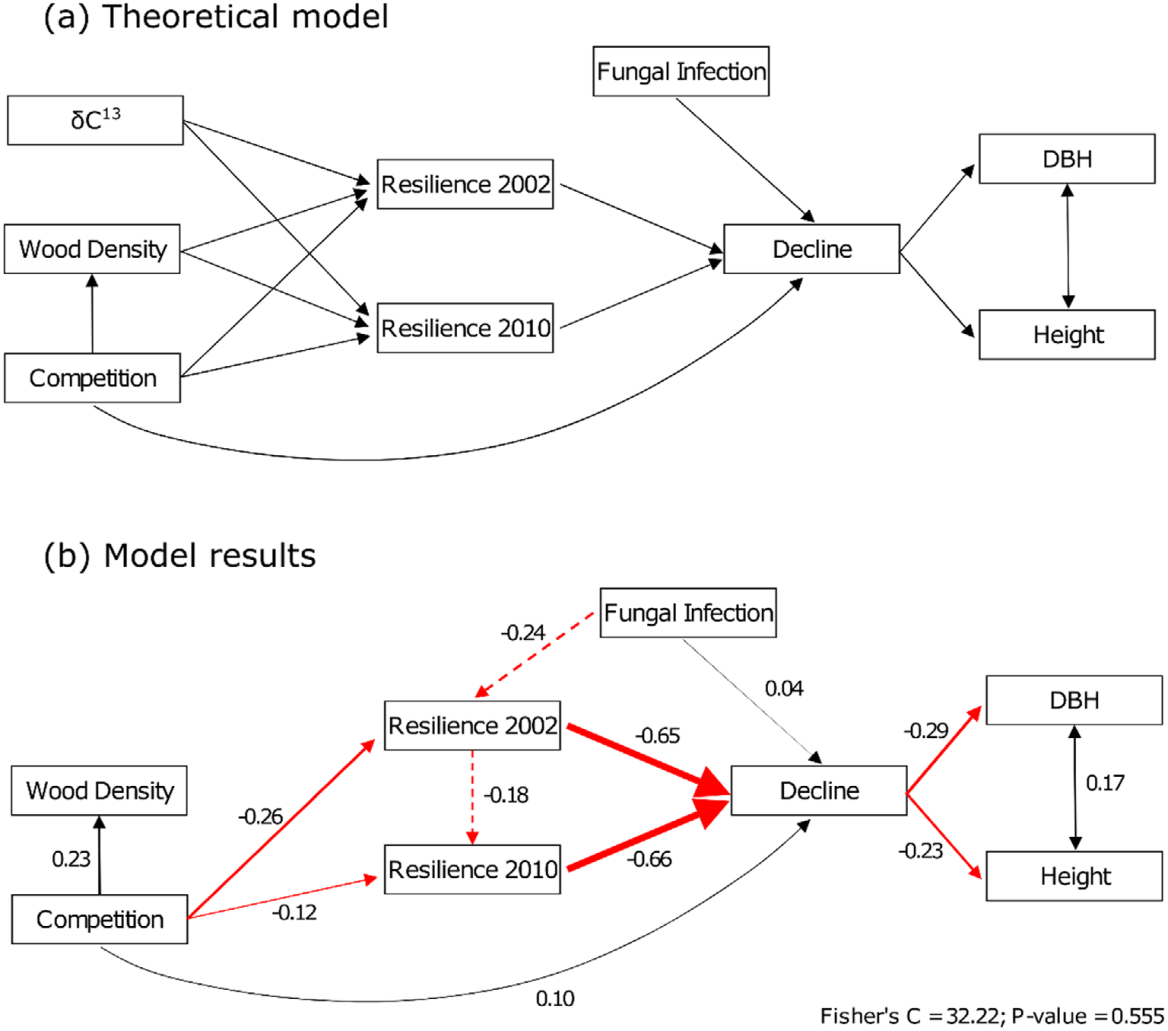
Representation of the structural equation model that relates the different physiological, drought response, biotic and abiotic stresses, and growth traits across four progeny trial test sites and 40 lodgepole pine families using drought response traits, fungal infection rating for western gall rust at age 36, height, and diameter at breast height (DBH) at age 35‐years (*N* = 1393). The theoretical model (a) was designed based on the expected relationships between traits. The model results (b) show which relationships were significant (black arrows for positive and red arrows for negative relationships) and the strength of the relationship (standardized coefficients and size of arrow). Dashed arrows were relationships that were not originally included in the model, but explained a significant part of the variance in the data.

We further explained the relationships between drought response indices, growth, physiological, and environmental variables with a piecewise SEM with a Fisher's *C* of 32.22 and *p* = 0.555 (Figure 5). Some of the trends identified in the previous section were also present in the SEM. Trees that were less drought resilient to either of the two drought events experienced higher long‐term decline which, in turn, reduced the trees' final growth as represented by height and DBH, which are correlated traits (Figure 5b). Although resilience to the 2002 and 2010 drought events was the variable with the greatest influence on decline, with standardized coefficients (SC) of −0.65 and −0.66, respectively, other stresses contributed, with a lower intensity, to the overall decline of the trees. Trees suffering from more competition had a stronger decline with an SC of 0.10, and trees suffering from a more severe infection of western gall rust also showed a higher decline (SC = 0.04). Trees suffering from more competition showed lower resilience to both drought events (SC = −0.26 and SC = −0.12 in 2002 and 2010, respectively), and trees with a greater presence of western gall rust appeared to be less resilient to drought in 2002 (SC = −0.24). Suppressed trees also showed higher wood density (SC = 0.23). We hypothesized, in our theoretical model, that δ^13^C, as a proxy for water use efficiency, and wood density, as a proxy for cavitation resistance, would favour drought resilience, but none of these relationships were found to be significant in the SEM.

### 3.3 Heritability and Genetic Control of Drought Adaptive Traits

All five drought response indices showed low to moderate heritability estimates (${\hat{h}}^{2}$) at each of the sites, while also showing relatively low estimates when all four sites were combined, indicating the presence of a G × E interaction (Table 3). Decline showed the highest estimates for the five drought response indices ranging from 0.15 to 0.30, across sites. Among the short‐term traits, resilience appeared to be a more heritable trait than resistance, despite VIRG 2010 resistance being the highest single heritability estimate (${\hat{h}}^{2}$= 0.39 ± 0.06) (Table 3). Growth traits showed higher heritability estimates than drought response traits, both at the individual site and combined site level, with height being the most heritable trait (${\hat{h}}^{2}$= 0.40–0.81) followed by DBH (${\hat{h}}^{2}$= 0.20–0.64) (Table 3). The lower estimates in the combined site data were due to G × E, as evidenced by the poor genetic correlations between sites (Table S1). Most traits were not significantly correlated between sites (Table S1). SWAN and TIME, however, the sites with the most similar environmental conditions (Table 1), showed the highest correlations in decline (0.81), resilience 2002 (0.73), and resistance 2002 (0.78). The only other significant correlation was between JUDY and SWAN for resilience 2010 (0.73).

**TABLE 3:** Narrow sense heritabilities (±SE) of the five drought response indices, height, and diameter at breast height (DBH, 1.3 m) measured at age 35 years of the tested lodgepole pine trees, calculated from the combined data from all sites combined, and each site individually (JUDY = Judy Creek, SWAN = Swan Hills, TIME = Timeau, VIRG = Virginia Hills).

Trait	All sites	JUDY	SWAN	TIME	VIRG
Decline	0.12 (0.03)	0.30 (0.12)	0.24 (0.04)	0.30 (0.08)	0.15 (0.02)
Resilience 2002	0.09 (0.03)	0.27 (0.11)	0.29 (0.04)	0.15 (0.06)	0.07 (0.01)
Resilience 2010	0.06 (0.02)	0.08 (0.05)	0.25 (0.03)	0.21 (0.06)	0.25 (0.04)
Resistance 2002	0.06 (0.02)	0.19 (0.08)	0.16 (0.02)	0.17 (0.07)	0.03 (0.01)
Resistance 2010	0.03 (0.01)	0.12 (0.08)	0.03 (0.01)	0.11 (0.05)	0.39 (0.06)
DBH 35	0.29 (0.05)	0.47 (0.14)	0.22 (0.03)	0.20 (0.06)	0.64 (0.08)
Height 35	0.50 (0.06)	0.54 (0.15)	0.81 (0.08)	0.50 (0.14)	0.40 (0.06)

Significant genetic correlations between traits followed a similar trend as the phenotypic correlations with both the combined site (Figure 4) and individual site analyses (Figures S4 and S5). Resilience, to both 2002 and 2010 events, was significantly correlated to the long‐term decline (*R* = −0.78 and −0.82, respectively; Figure 4b). The decline was also strongly correlated to both growth traits (*R* = −0.62 height and *R* = −0.55 DBH). Furthermore, height was significantly correlated to resilience 2010 (*R* = 0.57) and DBH was correlated to resilience 2002 (*R* = 0.60) (Figure 4b).

**FIGURE 6:**
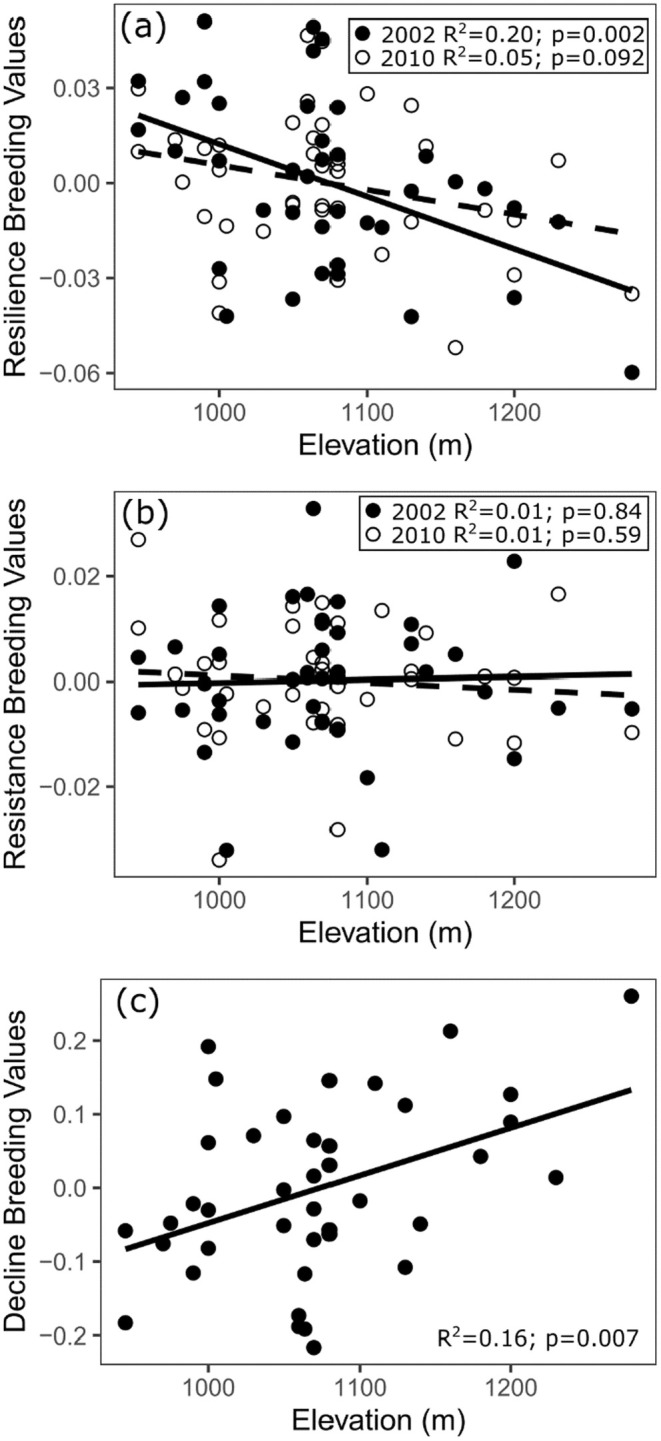
Relationships between the elevation of parent tree origin (provenance) of the lodgepole pine seeds and the breeding values of resilience (a), resistance (b), and decline (c). For the resilience and resistance indices, closed circles and solid lines represent the values for the 2002 drought event, and open circles and dashed lines represent the 2010 drought event.

Finally, we observed an adaptation to the local environment in resilience to the 2002 drought event and long‐term decline. Both traits were significantly correlated with the elevation of origin (provenance) of the donor mother trees (*R* ^2^ = 0.20, *p* = 0.002 for resilience 2002 and *R* ^2^ = 0.16, *p* = 0.007 for decline; Figure 6). Trees that originated from lower elevations with higher temperatures and lower precipitation (i.e., drier conditions) showed an increased drought response.

## 4 Discussion

This study analyzed the drought resilience and resistance of 40, 35‐year‐old lodgepole pine OP families growing at four progeny test sites that experienced multiple drought events. Under frequent drought events in the second half of the growing period, trees exhibiting high short‐term drought resilience (up to four years after the drought event) showed lower long‐term growth decline, and achieved superior final height and diameter at breast height (DBH). This positive relationship between drought resilience and final growth is in contrast to the more common observations found where there is a trade‐off between drought response and tree size (Bennett et al. 2015; Merlin et al. 2015; Serra‐Maluquer et al. 2018). Earlier studies suggested that larger trees were typically more susceptible to drought stress, and this was attributed to higher carbon allocation to aboveground biomass, higher hydraulic vulnerability, higher evaporative demands due to their stand's social status, and the preference of bark beetles to attack larger trees (Serra‐Maluquer et al. 2018). However, contrary findings have also been reported, indicating that larger trees may have a substantial root system, which favours water acquisition during drought (Hember et al. 2017; Zang et al. 2012). The abovementioned studies considered drought response to tree size. In this study, we have shown that trees have the ability to recover from a single or multiple drought events in early life stages, which in turn can affect a tree's long‐term growth. In fact, we found little to no relationship between the response to one or multiple drought events and tree growth prior to the drought event(s) (Figure 3, Figure S2). Our results show how drought‐intolerant trees can experience a severe long‐term reduction in growth if drought events continue to increase in frequency and severity, leading to increased tree mortality, and ultimately impacting the global carbon cycle in the face of climate change (Anderegg et al. 2015).

If including drought adaptation as a priority in forest management is desired, knowledge about the mechanisms underlying drought adaptation in both the short‐ and long‐term is essential. Our results demonstrate the greater impact of resilience on long‐term growth and decline than resistance (Figure 4). This prevalence of resilience as a drought adaptation strategy has previously been reported in maritime pine ( *Pinus pinaster* Ait.) provenances adapted to drier conditions (Sánchez‐Salguero et al. 2018). Higher resilience has been shown to be associated with lower drought‐induced mortality in conifers, as highlighted in a global study, contrasting with the greater influence of drought resistance in angiosperms (DeSoto et al. 2020). In a climate change scenario, characterized by recurring mild to severe drought events, such as the climate experienced in this study, the ability for rapid recovery after drought stress becomes critical in preventing long‐term growth decline. An alternative hypothesis is that lower resilience, as measured by recovery to pre‐drought growth levels, may indicate a plastic adaptation to drought. This plasticity could involve trees sacrificing aboveground growth in exchange for developing drought‐resistant tissues and a larger root system (Corcuera et al. 2004; Eilmann et al. 2009; Gessler et al. 2020; Hagedorn et al. 2016).

Furthermore, our study revealed a negative correlation between resistance and resilience at the phenotypic level in the two drought events studied, lending support to the hypothesis that a slow recovery from one drought event may lead to increased plasticity in drought response to subsequent drought events (Reed et al. 2011). However, it is also worth noting alternative interpretations of these results. First, the strong negative genetic correlation between resilience to the 2002 drought event and long‐term decline (*R* = −0.78; *p* < 0.001, Figure 4b) indicates that 14 years after the drought event, trees still struggled to recover to their pre‐drought growth rates. Other studies on drought‐induced mortality have shown a similar declining trend in growth for over 20 years before tree death, emphasizing the importance of maintaining consistent growth rates in order to survive future drought events (Camarero et al. 2015; DeSoto et al. 2020). A second consideration is that the moderately positive genetic correlation between resilience to both the 2002 and 2010 drought events (*R* = 0.40; SE = 0.22, Figure 4b), though not statistically significant, suggests that drought‐adapted trees generally showed better resilience to multiple drought events. The observed negative phenotypic correlation can be explained by extreme cases where trees that overcompensated their growth after the 2002 drought (with resilience values higher than 1) were severely affected by the 2010 event, or trees that did not recover after 2002 and maintained a low growth rate (i.e., high resilience) after the 2010 drought event.

Considerable research efforts have been invested in finding adaptive traits linked to drought responses, including associations with traits such as high wood density and specific leaf area (Greenwood et al. 2017), or higher plasticity in xylem anatomy and water use efficiency (Isaac‐Renton et al. 2018). Furthermore, higher wood density, for example, is often linked to tracheids with smaller lumen areas and thicker cell walls, providing a measure of resistance to embolism under drought stress (Hacke et al. 2001; Sperry et al. 2006). While we initially hypothesized that lodgepole pine trees with higher wood density would demonstrate greater drought resistance and resilience, our results did not support this expectation (Figure 5), further confirming the results obtained by Da Ros et al. (2021), using the same dataset. Weak associations between wood density and drought resistance and resilience have also been seen in *Abies* species (George et al. 2015), showing that xylem properties may not be the most relevant trait for assessing drought adaptation in conifers. Choat et al. (2012) suggest that most forest tree species operate at low hydraulic safety margins (i.e., the difference between common water potentials in the xylem and minimum water potential that would cause xylem damage), independent of xylem properties or climatic conditions. This implies that the ability to regulate stomatal closure and increase water use efficiency during drought events could be key in lodgepole pine. Isaac‐Renton et al. (2018) found that drought‐adapted lodgepole pine provenances could adjust stomatal behaviour under drought conditions, allowing these provenances to achieve higher final growth. Although we did not identify a correlation between trees' drought response and water use efficiency, as measured by δ^13^C, this data was derived from an average of the entire growing period captured by wood cores taken at breast height (approximately ages 20–35), making it a challenge to discern a potential plastic response to specific drought events.

Extrinsic factors, beyond the drought event itself, were also found to influence the trees' ability to resist and recover from drought stress. Our study observed that competition and susceptibility to western gall rust reduced drought resilience and increased long‐term decline values (Figure 5). While smaller trees may have experienced less drought stress due to shelter provided by more dominant trees (Aussenac 2000), being outcompeted for resources could hinder their ability to recover after a drought event. Competition can have a comparable or even greater impact than climate on long‐term growth trends (Gleason et al. 2017; Young et al. 2017; Zhang et al. 2015), however, it can also have a strong effect on short‐term recovery after drought. In our study, the two sites with ~50% mortality before the first drought event, and therefore lower competition, showed significantly higher resilience and lower decline. This observation suggests that managing for wider spacing (more open stands), with reduced competition, could be a strategy to mitigate the impact of drought on tree growth, fostering drought‐resilient forests (D'Amato et al. 2013; Sohn et al. 2013, 2016).

Abiotic (e.g., drought) and biotic (e.g., disease) stresses can interact synergistically, serving to predispose and incite factors, resulting in long‐term decline or mortality (McDowell et al. 2008). Our results show that trees heavily infected by western gall rust exhibited a diminished drought response. The negative impact of western gall rust on the hydraulic properties of lodgepole pine was studied by Wolken et al. (2009), suggesting that, under wet conditions, affected trees had lower hydraulic capacity while both infected and healthy trees were equally impacted by drought. This observation further explains why trees affected by western gall rust appeared compromised in their ability to recover to pre‐drought growth levels compared to healthy trees, as they may not have been able to take full advantage of periods with adequate water availability.

Several recent conifer studies have analyzed genetic adaptation to drought using dendrochronological traits. Some studies show linkages between dendrochronological traits and functional molecular markers (Heer et al. 2018; Housset et al. 2018; Trujillo‐Moya et al. 2018), while others report strong adaptation to local conditions when seed sources were from drier provenances (Depardieu et al. 2020; Montwé et al. 2016). These studies highlight the potential of using tree‐ring‐based traits, such as resilience, in genomic selection. Here, we found low to moderate heritability estimates in single‐site genetic models, with comparatively lower heritability from all sites combined (Table 3). In all cases, the heritability estimates of the five drought response traits were lower than the classic growth traits, such as height and DBH. Similar heritability values in short‐term indices at a single site were also reported in a recent white spruce ( *Picea glauca* (Moench) Voss) study (Depardieu et al. 2020). The lower heritability estimates in combined site models were explained by the low correlation between sites (i.e., G × E). Tree‐ring‐based indices reflect a tree's reaction to a specific environmental condition, contributing to the variability of drought response across sites, even for the same genotypes (Zas et al. 2020). For instance, a site with a deep rooting zone can favour genotypes that prioritize root growth and are capable of accessing water stored from deeper soil layers, while a dense stand might favour trees with fast above‐ground growth that can quickly outcompete their neighbours (Kozovits et al. 2005; Warren et al. 2005). Furthermore, how trees react to one drought event may affect how they react to subsequent drought events, introducing an additional environmental impact with the presence of a correlated response. If drought events occur at a high frequency, a cumulative effect could devalue the use of a single resilience index as a drought response trait. Despite exposure to two similar drought events at four progeny test sites within a single breeding region (i.e., “uniform” environmental conditions), we observed low correlations between sites or drought events (Table 1, Table S1), similar to Zas et al. (2020), where different responses were observed depending on the site or drought event. With these findings, we propose a new decline index that could reduce the cumulative effect of interactions between multiple consecutive drought events and provide a more reliable estimation of drought response in even‐aged common garden‐type trials. Our decline index exhibited higher and more stable heritability estimates across sites than classic drought response indices (Table 3), and showed the highest correlation between sites (Table S1). The decline was also one of the two drought response indices to show significant adaptation to the local environment of the parent trees (Figure 6).

Several studies have demonstrated local adaptation to drought in conifers using dendrochronological traits (Depardieu et al. 2020; Montwé et al. 2016) or other approaches (de la Mata et al. 2014; Matías et al. 2014; Voltas et al. 2008). These studies, showing local adaptation, have been used to advocate for assisted migration between breeding regions as a tool to achieve drought‐adapted forests (Aitken and Whitlock 2013; Pedlar et al. 2012). In our study, we observed local adaptation of lodgepole pine families originating from lower elevations showing better resilience and lower decline than families from higher elevations. Detecting local adaptation within a range of 60 km in distance and only 300 m in elevation is remarkable, considering the relatively low environmental variation this captures, compared to other studies on local adaptation (Depardieu et al. 2020; Montwé et al. 2016; Zas et al. 2020). These results underscore the potential of seed transfers from drier to wetter areas to improve the drought response of a seed source, even within a single breeding region, when assisted migration from outside the breeding region is not desirable.

## 5 Conclusions

Our results show that short‐term drought response is a primary driver of the long‐term performance of trees exposed to multiple drought events. With the anticipated increase in drought frequency, trees with drought resilience are likely to grow faster in contrast to the commonly observed trade‐off between size and drought response. Furthermore, a structural equation model revealed that a negative effect of fungal infection, combined with competition, can impact a tree's drought response.

Dendrochronological traits are promising for the selection of drought‐adapted genotypes. We found evidence of genetic control in the growth response to drought, with moderate heritability estimates in several drought response indices, which was partially attributed to local adaptation to drier environments. However, there was substantial variability in the response of trees from the same families to different drought events across growing sites.

Lastly, we introduced a new decline index, providing a better indicator of a drought response compared to traditional dendrochronology indices. This new index is useful when multiple drought events occur over a short period of time, as it captures the long‐term effect on growth trends. The decline index has higher heritability and shows a stronger correlation with growth traits, making it a promising candidate trait for the selection of drought‐adapted genotypes in tree improvement programs.

## Author Contributions

**Jaime Sebastian‐Azcona:** conceptualization (equal), data curation (lead), formal analysis (lead), funding acquisition (supporting), investigation (lead), methodology (lead), project administration (supporting), resources (supporting), software (supporting), supervision (supporting), validation (equal), visualization (equal), writing – original draft (lead), writing – review and editing (supporting). **Eduardo P. Cappa:** formal analysis (equal), methodology (equal), validation (equal), visualization (equal), writing – original draft (supporting), writing – review and editing (supporting). **Letitia Da Ros:** data curation (supporting), formal analysis (equal), methodology (supporting), validation (supporting), writing – review and editing (supporting). **Blaise Ratcliffe:** conceptualization (supporting), formal analysis (equal), software (supporting), validation (equal), visualization (supporting), writing – review and editing (supporting). **Charles Chen:** conceptualization (supporting), formal analysis (equal), funding acquisition (supporting), investigation (supporting), project administration (supporting), supervision (supporting), validation (supporting), visualization (supporting), writing – original draft (supporting), writing – review and editing (equal). **Xiaojing Wei:** data curation (equal), formal analysis (supporting), validation (equal), visualization (equal), writing – original draft (supporting), writing – review and editing (supporting). **Yang Liu:** formal analysis (equal), methodology (supporting), validation (supporting), visualization (supporting), writing – review and editing (supporting). **Shawn D. Mansfield:** data curation (equal), funding acquisition (supporting), methodology (supporting), validation (supporting), writing – review and editing (supporting). **Andreas Hamann:** conceptualization (supporting), funding acquisition (supporting), methodology (supporting), supervision (supporting), writing – original draft (supporting), writing – review and editing (equal). **Yousry A. El‐Kassaby:** conceptualization (supporting), formal analysis (supporting), funding acquisition (equal), project administration (equal), supervision (supporting), writing – review and editing (supporting). **Barb R. Thomas:** conceptualization (equal), data curation (equal), funding acquisition (lead), investigation (equal), methodology (supporting), project administration (lead), resources (equal), supervision (lead), validation (supporting), visualization (supporting), writing – original draft (supporting), writing – review and editing (lead).

## Conflicts of Interest

The authors declare no conflicts of interest.

## Supporting information


Data S1

## Data Availability

Data associated with this study is available at https://doi.org/10.6084/m9.figshare.c.7575752.
