# Supplementary material for: Short‐ and Long‐Term Growth Response to Multiple Drought Episodes: Evidence of Genetic Adaptation in a Conifer Species
Source: Ecol Evol. 2025 May 14;15(5):e71398. doi: 10.1002/ece3.71398 (PMC12076065; doi:10.1002/ece3.71398)
Supplement: Supplementary file 1 — Data S1 [file ECE3-15-e71398-s001.docx]

**Supplementary material**

**Table S1.** Additive genetic correlations between the four lodgepole pine progeny trial test sites for each of the five drought response traits. The approximated standard error of the correlation is given in parenthesis. Where JUDY = Judy Creek, SWAN = Swan Hills, TIME = Timeau, VIRG = Virginia Hills test sites in Alberta.

| Decline | JUDY | SWAN | TIME | VIRG |
| --- | --- | --- | --- | --- |
| JUDY | 1 |  |  |  |
| SWAN | 0.29 (0.22) | 1 |  |  |
| TIME | 0.16 (0.24) | 0.81 (0.10) | 1 |  |
| VIRG | 0.26 (0.28) | 0.53 (0.43) | 0.36 (0.34) | 1 |

| Resilience 2002 | JUDY | SWAN | TIME | VIRG |
| --- | --- | --- | --- | --- |
| JUDY | 1 |  |  |  |
| SWAN | 0.34 (0.31) | 1 |  |  |
| TIME | -0.24 (0.52) | 0.73 (0.21) | 1 |  |
| VIRG | -0.58 (0.87) | 0.16 (0.69) | 0.46 (0.77) | 1 |

| Resilience 2010 | JUDY | SWAN | TIME | VIRG |
| --- | --- | --- | --- | --- |
| JUDY | 1 |  |  |  |
| SWAN | 0.73 (0.24) | 1 |  |  |
| TIME | 0.01 (0.33) | -0.27 (0.28) | 1 |  |
| VIRG | 0.27 (1.50) | -0.01 (0.80) | 0.57 (0.32) | 1 |

| Resistance 2002 | JUDY | SWAN | TIME | VIRG |
| --- | --- | --- | --- | --- |
| JUDY | 1 |  |  |  |
| SWAN | 0.82 (0.52) | 1 |  |  |
| TIME | 0.52 (0.33) | 0.78 (0.31) | 1 |  |
| VIRG | 0.02 (1.15) | 0.19 (1.01) | 0.48 (0.68) | 1 |

| Resistance 2010 | JUDY | SWAN | TIME | VIRG |
| --- | --- | --- | --- | --- |
| JUDY | 1 |  |  |  |
| SWAN | 0.54 (0.69) | 1 |  |  |
| TIME | -0.43 (2.36) | -0.19 (0.82) | 1 |  |
| VIRG | -0.72 (0.54) | -0.18 (0.53) | 0.49 (0.51) | 1 |


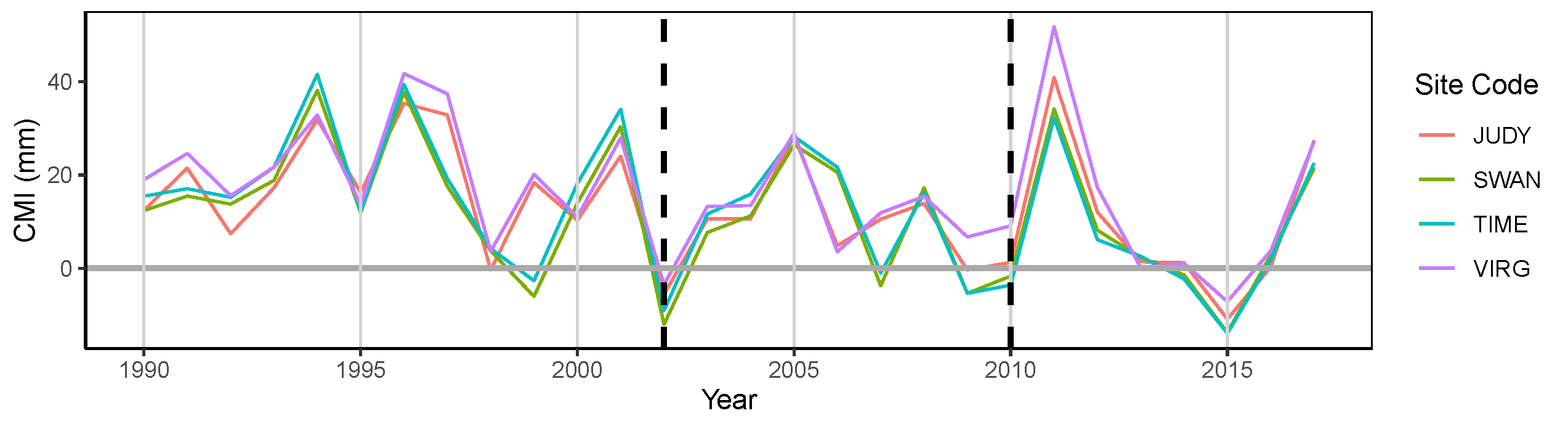

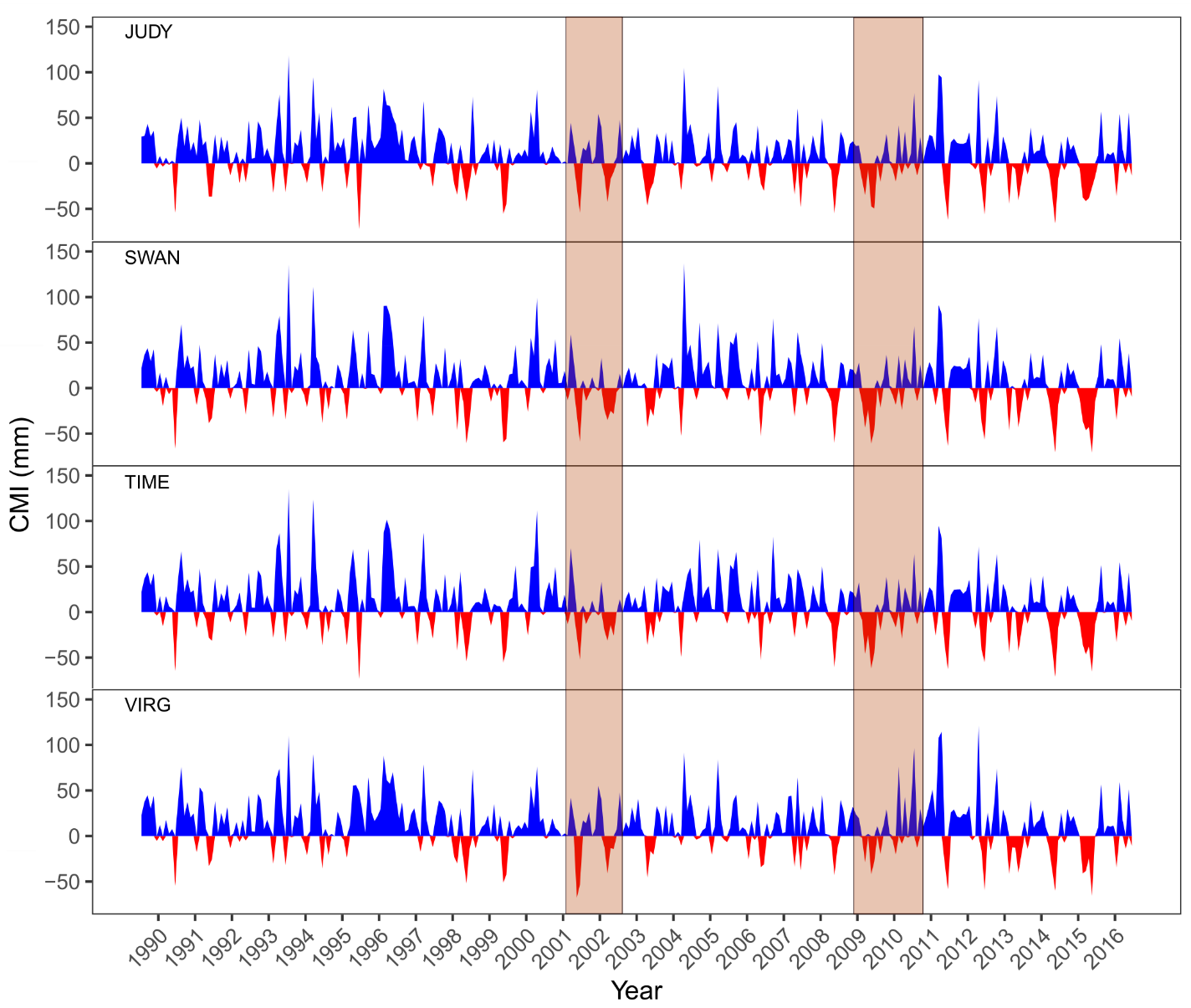


**Figure S1.** Monthly (top 4 panels) and annual (bottom panel) climate moisture index (CMI) values observed at the four test sites (JUDY = Judy Creek, SWAN = Swan Hills, TIME = Timeau, VIRG = Virginia Hills) during the growing period of the plantations. Annual CMI values were calculated from the end of the previous growing season (September) to the end of the current growing season (August). Vertical dashed lines indicate when a drought response was observed in tree ring data in 2002 and 2010.


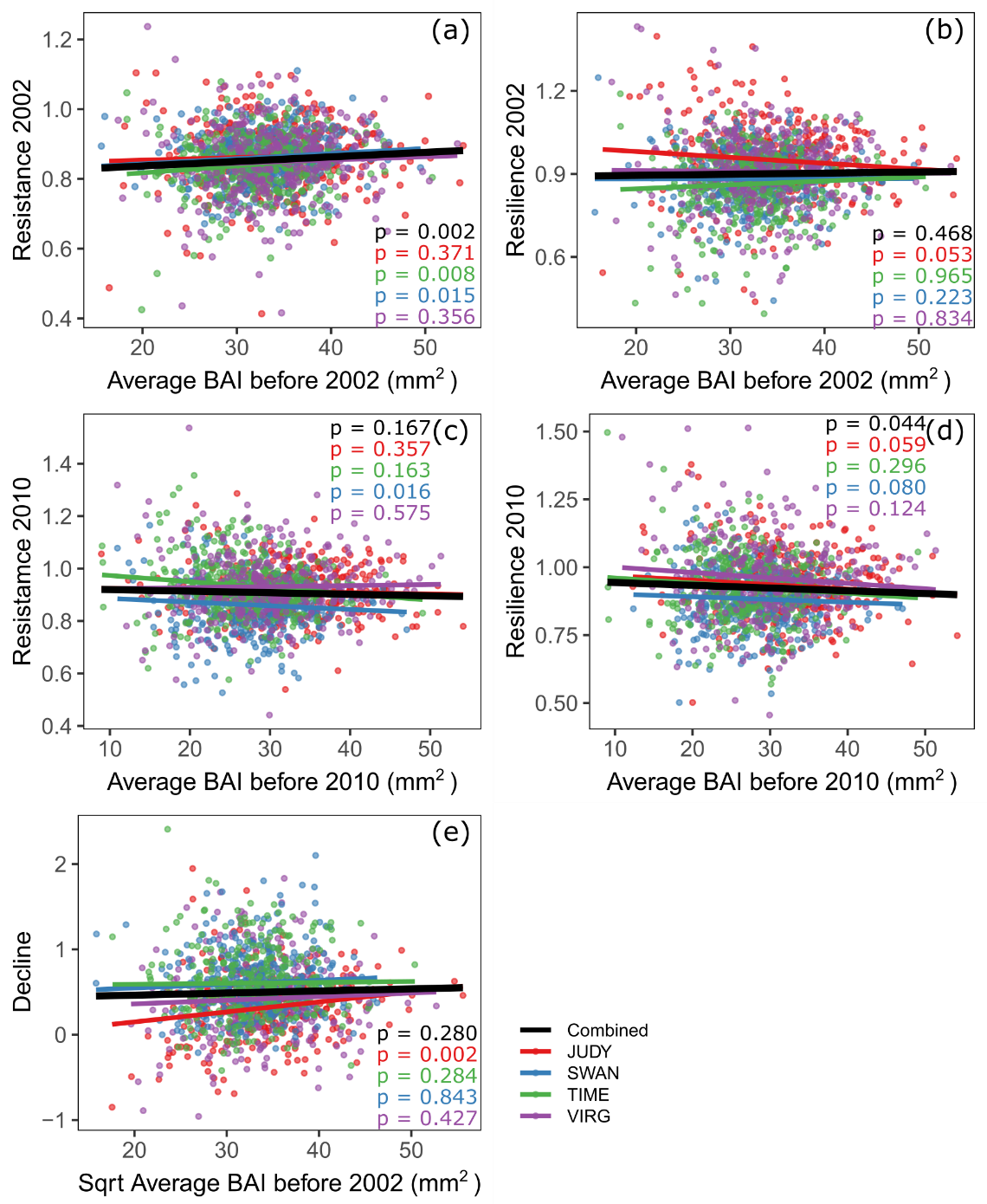


**Figure S2.** Relationship between resistance (a, c), resilience (b, d), and decline (e), and the average growth of the four years prior to the 2002 (a, c) and 2010 (b, d) drought events for the resistance and resilience indices, and the average growth during the five-year period of maximum growth for decline at each of the four lodgepole pine progeny trial test sites in Alberta (JUDY = Judy Creek, SWAN = Swan Hills, TIME = Timeau, VIRG = Virginia Hills).


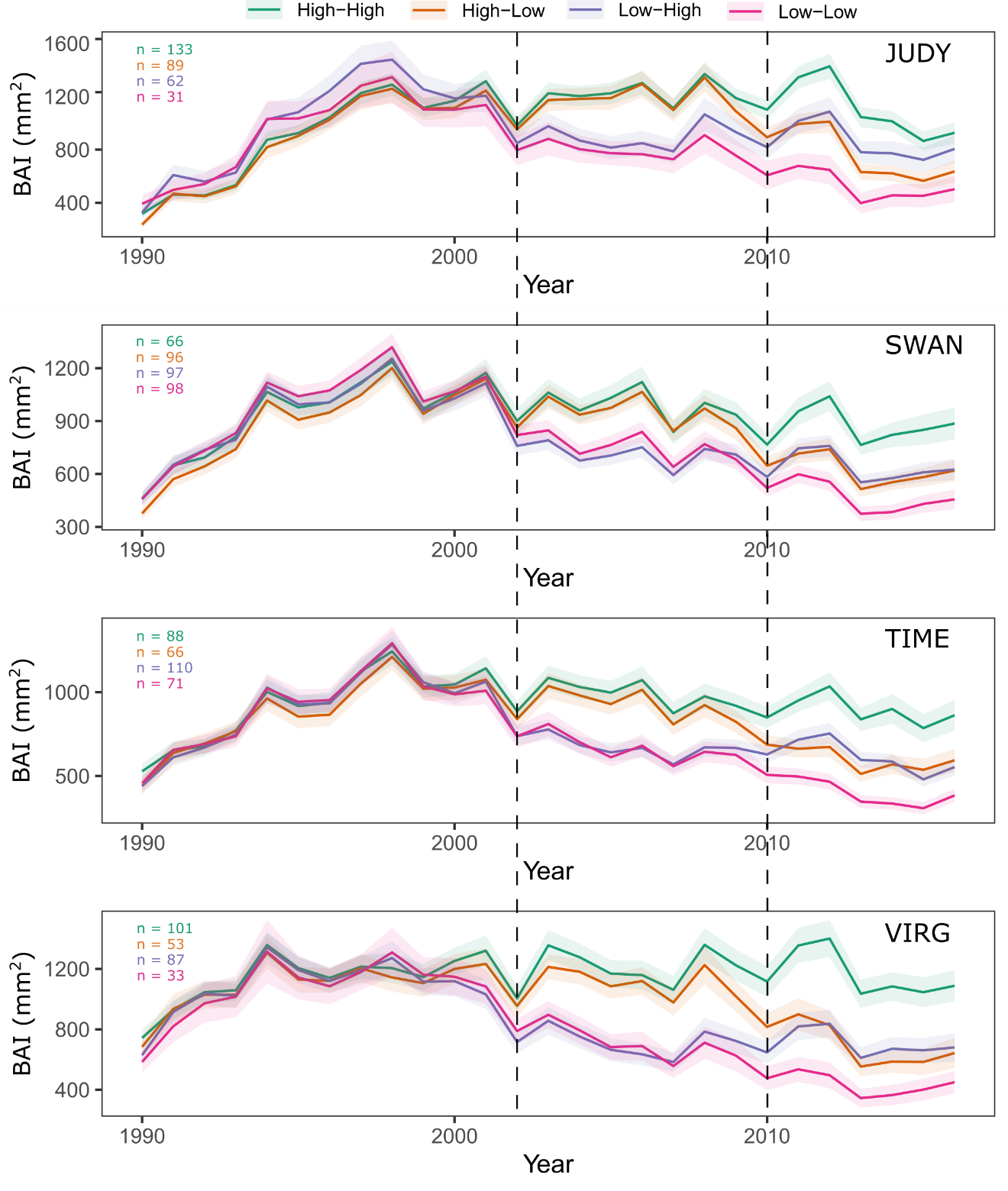


**Figure S3.** Average yearly basal area increment (BAI) of the lodgepole pine trees divided by the four progeny trial test sites and drought resilience groups (a). The High-High group represents trees that showed good resilience (>0.8) in both the 2002 and 2010 drought events (dashed vertical lines), High-Low and Low-High groups showed good resilience in only one drought event, and the Low-Low group showed poor resilience after both drought events. Where JUDY = Judy Creek, SWAN = Swan Hills, TIME = Timeau, VIRG = Virginia Hills test sites in Alberta.


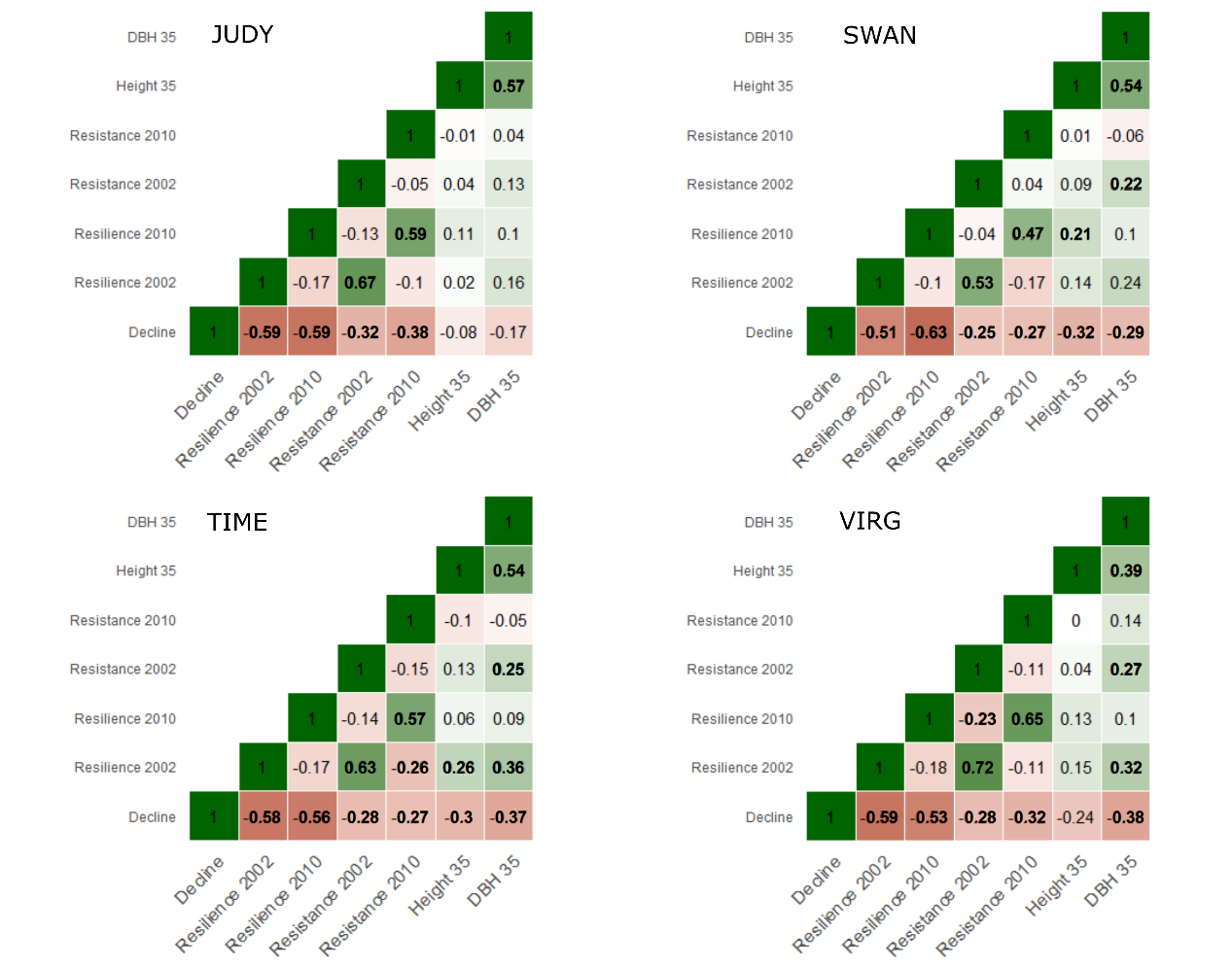


**Figure S4.** Phenotypic correlations of 40 lodgepole pine families across four progeny trial test sites (JUDY = Judy Creek, SWAN = Swan Hills, TIME = Timeau, VIRG = Virginia Hills), between drought response indices, height, diameter at breast height (DBH, 1.3m) at age 35-years (N = 1,393). Significant phenotypic correlations after a Bonferroni adjustment for multiple inference (α = 0.05) are highlighted in bold.


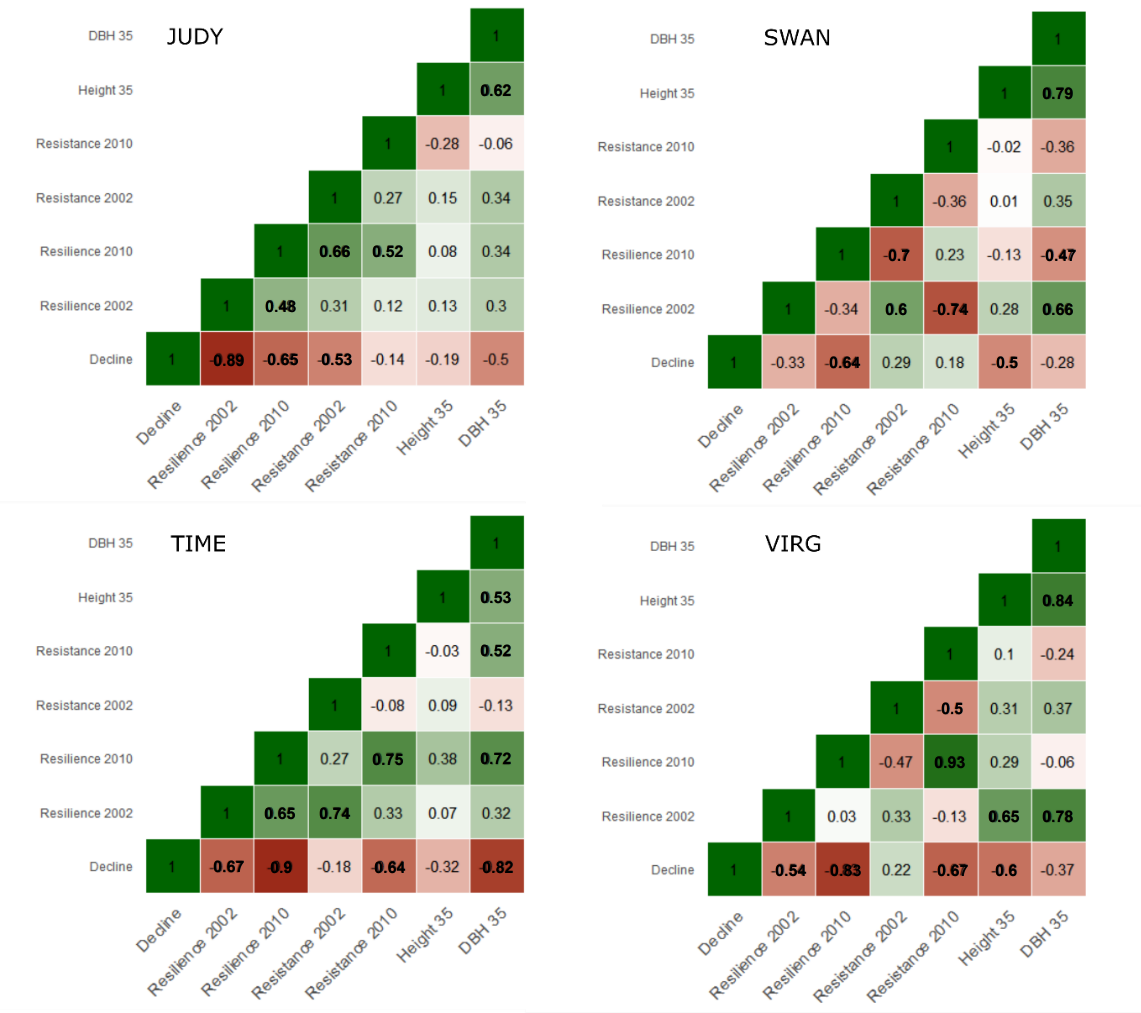


**Figure S5.** Additive genetic correlations of 40 lodgepole pine families across four progeny test sites (JUDY = Judy Creek, SWAN = Swan Hills, TIME = Timeau, VIRG = Virginia Hills), between drought response indices, height, diameter at breast height (DBH, 1.3m) at age 35-years (N = 1,393). Significant genetic correlations after a Bonferroni adjustment for multiple inference (α = 0.05) are highlighted in bold.
